# Predicting binge drinking among university students: Application of integrated behavioral model

**DOI:** 10.1371/journal.pone.0254185

**Published:** 2021-07-09

**Authors:** Hordofa Gutema, Yamrot Debela, Bizuayehu Walle, Kidist Reba, Tebkew Shibabaw, Tolera Disasa

**Affiliations:** 1 Department of Health Education, School of Public Health, College of Medicine and Health Science, Bahir Dar University, Bahir Dar, Ethiopia; 2 Department of Physiology, School of Medicine, College of Medicine and Health Science, Bahir Dar University, Bahir Dar, Ethiopia; 3 Department of Adult Health Nursing, School of Nursing, College of Medicine and Health Science, Bahir Dar University, Bahir Dar, Ethiopia; 4 Department of Environmental Health, School of Public Health, College of Medicine and Health Science, Bahir Dar University, Bahir Dar, Ethiopia; 5 Marie Stopes Ethiopia, Head Office, Addis Ababa, Ethiopia; Harvard Medical School, UNITED STATES

## Abstract

**Background:**

Binge drinking is a pattern of harmful use of alcohol and it is defined as four drinks for women and five drinks for men in about 2 hours. This behavior causes public health problems like damaging different body organs.

**Objective:**

To assess binge drinking and associated factors among Bahir Dar University students in Northwest Ethiopia.

**Method:**

A cross sectional study was conducted in November 2017. Systematic sampling technique was used to select 422 participants. Structured questionnaire was used to collect data. Linear and Logistic regression models were used to predict the role of explanatory variables on behavioral intention and binge drinking, respectively. Independent variables with a p-value of <0.05 at 95% confidence interval were considered as statistically significant in the final model.

**Result:**

A total of 413 students participated in this study and 33.4%(95% CI: 28.3–38.9) were engaged in binge drinking. Experiential attitude, instrumental attitude, and self-efficacy were found to be significant predictors of intention to binge drinking (p<0.05). Experiential attitude, environmental constraint, injunctive norm, and knowledge predictors were significantly associated with binge drinking (p<0.05).

**Conclusion:**

Our study indicated that one-third of the students practiced binge drinking. This behavior was associated with experiential attitude, injunctive norm, environmental constraints, and knowledge factors. Additionally, experiential attitude, instrumental attitude, and self-efficacy constructs had explained behavioral intention. This implies focusing on the abovementioned determinant factors is imperative while designing intervention strategy.

## Background

Alcohol is a substance which has the properties of psycho-activation and producing dependence among the user. Recent figures from the World Health Organization (WHO) reveal that the patterns of alcohol consumption range between continents and countries [1]. Harmful use of alcohol leads to undesirable public health (e.g. liver cirrhosis, cancers, and injuries) and socio-economic (e.g. family income draining, conflict)consequences in societies [2, 3]. Alcohol abuse is a causal factor for more than 200 diseases and injury conditions and contributes to 5.9% of deaths worldwide [4]. Despite its impact alcohol consumption has given relatively low importance in public policy, including in public health policy on alcohol use [1].

Binge drinking (BD) is considered as a harmful behavior and is defined as four drinks for women and five drinks for men in about 2 hours by bringing blood alcohol concentration levels to 0.08 g/dl [5]. Despite its impact on health, societies, and economy, drinking in students has become ceremonial and often has been seen as an essential part of their higher education experience [6]. Indeed, BD is a significant public health problem in many countries due to its high prevalence especially among young college students [7, 8]. BD during adolescence had been associated with disruption of brain development [9], increased risk of alcohol use disorder, and cardiovascular diseases [10]. Frequent BD for a long period of time also damages the liver and leads to disability and death [5]. BD can also cause serious safety risks, like car crashes, drunk-driving arrests, sexual assaults, and injuries [11].

Sales promotions like below cost and free drink, particularly by new brewery companies, have recently increased in Ethiopia. Such advertising of new products has high probability of creating new alcohol consumers and rising its patterns particularly in the younger generation like college students [12]. Additionally, although WHO highly recommends alcohol policy such as drink driving policy, legal minimum age for sales, displaying alcohol content on containers, legally obligatory regulations on alcohol advertising, pricing policy [1], these policies and regulations were not implemented in Ethiopia apart from displaying alcohol content on containers.

Integrated Behavioral Model (IBM) is the behavioral model which developed in 1990s for further extension of Theory of Planned Behavior (TPB). For both of these models, the most important determinant of behavior is intention perform the behavior; however, IBM includes 3 other constructs that are not utilized within the TPB. According to IBM, in addition to intention, a particular behavior is most likely to occur if; 1) a person has knowledge about, 2) there is no environmental constraint preventing performance, 3) a person has performed the behavior previously. The model also asserts, Direct determinants of individuals’ behavioral intention (BI) are their instrumental and experiential attitudes, injunctive and descriptive norms, self-efficacy, and perceived control [13]. To our knowledge, despite its better prediction by considering additional constructs, there is no apparent researcher who used IBM to investigate BD other than one study [14]. Although BD is reported to be common among university students, there is no study done in Ethiopia on university students. Therefore, the aim of this study is to assess BD, we used IBM as a conceptual framework to examine BD and associated factors among University students.

## Methods and materials

### Study area, design, and period

A cross sectional study was conducted among Bahir Dar University students from November 1–30, 2017. Bahir Dar University is located in Bahir Dar city, 565 kilometers away from Ethiopian capital, Addis Ababa.

### Population

All regular undergraduate students who were attending classes during the study period were considered in the study. Contrarily, students who were enrolled in any distance and extension program, and who were unable to communicate due to severe illness were excluded.

### Sample size and sampling procedure

Sample size was calculated using a single population proportion formula, assuming 95% confidence interval, 5% margin of error, and 50% proportion of BD. After adding 10% possible non-response rate, the sample size was determined to be 422 participants. To get individual participant, three academic units were randomly selected out of fifteen. Then, one department was again randomly selected from each of the selected academic unit and sample size was proportionally allocated to the departments based on the size of students. Sampling frame was developed by listing the students from the selected departments. Finally, systematic sampling procedure was applied to select participants from each department.

### Data collection procedure and quality assurance

Prior to developing the actual questionnaire, an elicitation study which is a critical step in applying IBM was conducted using an open-ended interview with 20 students. This is essential to identify relevant behavioral outcomes, referents, and environmental facilitators and barriers for BD. Based on the finding from elicitation study, IBM construct items were developed, and data was collected using self-administered structured questionnaire. Four diploma holder experienced data collectors and two supervisors were recruited for data collection. A two days training was given for data collectors and supervisors on how to collect the data and other related procedures.

### Instrument

#### Dependent variable

Binge drinking (measured by two questions).

#### Independent variables

These variables were comprised of Socio demographic characteristics (11 questions), Knowledge (13 items), Intention (4 items of five point likert scale), experiential attitude (5 items of five point likert scale), instrumental attitude(8 items of five point likert scale), injunctive norm (5 items of five point Likert scale), Descriptive norm (6 items of five point Likert scale), Perceived control(5 items of likely–unlikely scale), Self-efficacy (10 items of five point Likert scale) and environmental constraint (8 items). The detail definition of each variable is available as S2 File.

### Data processing and analysis

The data was entered into Epi data 3.1 and then exported, cleaned, and analyzed using SPSS version 21. Descriptive statistical analysis like frequency, percentage, mean and standard deviation were computed. The association between the intention and each construct of IBM was checked using Pearson’s correlation coefficient. Student t-test and chi-square were used as needed to check the association between each construct of IBM and intention to BD, and between knowledge, environmental constrain and BD. Linear regression model was run to identify predictors of intention to BD. Besides, logistic regression was done to identify the constructs of IBM that influence BD. Backward stepwise regression was used to fit the final model for both analysis. Prior to conducting the any of the regressions, multicollinearity test was done for both outcomes and homoscedasticity was checked for linear regression, and both of the tests has fulfilled the assumptions. The result of the beta coefficient and odds ratio was used for interpretation of strength of prediction of the independent variables to the outcomes. For all statistical significance tests, the cut- off value set was p<0.05 with 95% confidence interval.

### Ethics approval and consent to participate

The protocol of the study was reviewed and approved by the IRB of College of Medicine and Health Sciences, Bahir Dar University. Participant’s written informed consent was sought before they are recruited to participate in the study. Names and other personal information which can violate the confidentiality of the respondents were not taken. The data obtained in due course were confidentially stored.

## Results

A total of 413 students participated in this study with a response rate of 97.86%.

### Participants socio-demographic characteristics

The mean age of the participants was 23.2±1.6. Two hundred six (63.0%) of them were male and the majority (97.3%) of them were single. One hundred eighty-eight (45.5%) of the participants were from textile engineering academic units. Majority (80.1%) of them were living in the campus. Regarding the educational status of their family, 28.8% and 39.5% of the participants mother and father have completed college, respectively. Around two third of the participants’ families were living in urban areas as presented in Table 1.

**Table 1:** Socio-demographic characteristics of respondents (n = 413).

**Variable**	**Category**	**Frequency**	**Percent**
**Age (mean±SD)**	23.2 ±1.6		
**Gender**	Male	260	63.00
	Female	153	37.00
**Marital status**	Single	402	97.30
	Ever Married	11	2.70
**Academic unit**	Civil Engineering	165	40.00
	Textile Engineering	188	45.50
	Economics	60	14.50
**Year of study**	First	12	2.90
	Second	49	11.90
	Third	118	28.60
	Fourth	58	14.00
	Fifth	176	42.60
**Living condition**	With parent	82	19.90
	In a campus	331	80.10
**Educational status of mother**	Illiterate	71	17.20
	Able to read and write	101	24.50
	Elementary school	41	9.90
	Secondary school	81	19.60
	College	119	28.80
**Educational status of father**	Illiterate	55	13.30
	Able to read and write	98	23.70
	Elementary School	36	8.80
	Secondary School	61	14.70
	College	163	39.50
**Family place of residence**	Urban	261	63.20
	Rural	152	36.80

### Alcohol drinking, binge drinking and knowledge

Around three-fourth (75.3%) of the students had alcohol drinks in their lifetime. The commonest (84.6%) type of alcohol drink consumed by the participants was beer. The average number of alcoholic drinks consumed by participants the last time they socialized was 4.4 (SD± 3.6) and the average time spent on drinking alcoholic beverages was 2.8 hours (SD ± 1.8). Including those who are abstaining, one-fourth (25.2%) of the participants were engaged in BD in the last month. In addition, BD magnitude was found 33.4% (95% CI: 28.3–38.9) when those who abstain are excluded. The participants reported that it took them an average of six drinks (SD± 3.5) to become drunk. Two hundred sixty (63%) of the participants have never heard about BD. Around one-third (32.7%) of them were knowledgeable about BD, Table 2.

**Table 2:** Alcohol drinking pattern, binge drinking and knowledge of participants (n = 413).

Variable	Frequency	Percent	Mean±SD
**Ever had any alcoholic drink**			
No	102	24.70	
Yes	311	75.30	
**Type of alcohol drink consumed**			
Beer	263	84.60	
Wine	18	5.80	
Distilled	10	3.20	
Sprit	5	1.60	
Other	15	4.80	
**Number of alcoholic drinks consumed the last time socialized**	311		4.4±3.6
**Hours spent drinking**	311		2.8±1.8
**Binge drinking**			
No	207	66.60	
Yes	104	33.40	
**Number of drinks to become drunk**	311		6.2±3.5
**Ever heard of about BD**			
No	260	63.00	
Yes	153	37.00	
**BD knowledge**			
Not knowledgeable	278	67.30	
Knowledgeable	135	32.70	

### Association between behavioral intention to binge drinking and constructs of IBM

Linear regression analysis was performed by using behavioral intention (BI) as outcome variable and the six constructs of IBM as explanatory variable. This model has explained 41.2% (Adjusted R^2^ = 41.2%) of the variance for BI to BD. Experiential attitude (B = 0.28, P< 0.001) and instrumental attitude (B = 0.22, P< 0.001) have positive significant association with BI. This implies that for a unit positive change in experiential attitude and instrumental attitude, the intention to BD will increase by 0.28 and 0.22 respectively. Oppositely, self-efficacy was negatively associated with BI (B = -0.26, P< 0.001). It appeared that a unit decrease of self-efficacy toward preventing BD, there will be an increase in BI toward BD by 0.26, Table 3.

**Table 3:** Behavioral intention and its association with constructs of IBM among participants.

IBM constructs	Mean	SD	Beta	p-value	95% CI	
					Lower	Upper
**Experiential attitude**	16.64	4.6	0.28	p<0.001	0.14	0.28
**Instrumental attitude**	27.54	5.06	0.21	p<0.001	0.09	0.22
**Injunctive norm**	15.84	3.5	0.03	0.59	-0.07	0.12
**Descriptive norm**	21.72	4.02	0.09	0.05	-0.01	0.17
**Perceived control**	49.52	8	0.01	0.92	-0.02	0.06
**Self-efficacy**	27.38	5.44	-0.26	p<0.001	-0.22	-0.11

### Predictors of binge drinking among university students

Multiple logistic regression was performed to predict the effect of IBM constructs on BD. This model captured 37.4% (Nagelkerke pseudo R^2^) of the variance in BD. Experiential attitude (AOR = 1.16, 95% CI [1.08–1.25]) found to have positive significance association with BD. It implies that a unit increase in experiential attitude toward BD, the odds of BD increase by 0.16 unites. Environmental constraint was another construct which has positive significant association with BD at (AOR = 1.31, 95% CI [1.06–1.60]). This indicates that as environmental constraints for refraining from BD increase by one, the odds of student engagement in BD increase 0.31 times. Injunctive norm (AOR = 0.89, 95% CI [0.81–0.98]) and Knowledge (AOR = 0.56. 95% CI [0.32–0.96]) had negative significant association with BD. It appeared that as the injunctive norm and knowledge decrease by one, the odds of BD increase by 11% and 44% respectively, Table 4.

**Table 4:** Predictors of binge drinking among participants.

Variable	Beta	Wald	Adjusted Odd Ratio	95% CI	
				Lower	Upper
**Behavioral intention**	-0.10	0.05	0.99	0.9	1.08
**Experiential attitude**	0.15	16.12	1.16	1.08	1.25
**Instrumental attitude**	0.02	0.15	1.01	0.94	1.09
**Injunctive norm**	-0.11	6.01	0.89	0.81	0.98
**Descriptive norm**	0.06	2.48	1.06	0.98	1.16
**Perceived control**	0.01	0.01	1.00	0.98	1.16
**Self-efficacy**	-0.03	1.93	0.96	0.92	1.01
**Knowledge**	-0.58	4.50	0.56	0.32	0.96
**Environmental constraint**	0.27	7.05	1.31	1.06	1.60

## Discussion

To our knowledge, the current study is conducted on BD for the first time in Ethiopia among University students. In this study, 33.4% of the students who drank alcohol were engaged in BD in the last one month. This finding is comparable with a study conducted among adolescents in Brazil as well as undergraduate university students in the USA, where 36% and 37% of the participants were engaged in BD, respectively [14, 15]. However, the current finding is lower than the one reported in a study conducted among adolescents in Germany, where the prevalence of BD was demonstrated to be 52.3% [16]. Difference in study setting might be the main reason for this discrepancy. Although the prevalence of BD in our study is found to be lower as compared to the above study, it is higher than the WHO report in which 16% of individuals who drank alcohol were engaged in BD worldwide [1]. An intervention that takes into account the negative consequence of BD on university students’ health should be implemented.

IBM explained 41.2% and 37.4% of variance for intention to BD and BD behavior in respective order in the multivariate analysis. It is comparable with a previous study which used IBM to predict BD among University students where the model had explained 44% and 26% of the variance intention and BD respectively [14]. However, this figure is lower than a study by Elliott & Ainsworth, where the theory of planned behavior had explained 90% of the variance in BD [17].

From six constructs of IBM, experiential attitude, instrumental attitude, and self-efficacy had statistically significant association with behavioral intention with beta coefficients of 0.28, 0.22, and -0.26, respectively. Experiential attitude is found to be the toughest positive determinant of behavioral intention, indicating that participants who have positive emotional experience are more likely intended to engage in BD in the future. In other studies, experiential attitude was significantly associated with intention to BD with strong positive beta coefficient [14, 17]. In a study conducted to assess testicular self-examination using IBM, experiential attitude was found to be a strong positive predictor of testicular self-examination. Although it was not as strong as experiential one, instrumental attitude was a positive significant predictor of intention to BD in our study. In another study, instrumental attitude had revealed the same significant beta coefficient with the current study [17].

The current study has also shown statistically significant association between self-efficacy and intention to BD with a negative beta coefficient, indicating inverse relationship. Hence, as a student’s self-efficacy decreased, intention to BD increased and vice versa. The findings from previous studies had also shown statistically significant negative association between self-efficacy and behavioral intention [14, 18]. On the other side, injunctive norm, descriptive norm, and perceived control constructs were not significantly associated with intention to BD. This indicates that referent others’ approval or belief in the presence of external control have no effect on intention to BD. The findings of earlier researches also reported mixed findings on these constructs. For instance, in a study by Braun *et al*., descriptive norm and perceived control were not significantly associated with intention, but injunctive norm [14], while a study by Elliott and Ainsworth had demonstrated that injunctive and descriptive norm constructs significantly associated with intention [17]. This inconsistency among the findings of the studies might be due to the difference of contexts in which behavior occurs and targets population.

Experiential attitude was found to be a positive significant predictor of BD in our study. Previously conducted study also supported this report [17]. Environmental constraint for refraining from BD was also found to have positive significant association with BD. This indicates that availability of grocery near the university; drinking specials like happy hour, and intensified alcohol advertisement of mass media were the environmental factors which increased student’s engagement in BD. Although environmental constraint was not significant construct for BD in a study which used IBM as conceptual framework [14], other reports showed that availability of alcoholic drink [19], alcohol establishments within certain area [20], and drinking special and promotions [12, 21] were identified as factors that affect drinking behavior.

Injunctive norm was found to have a negative significant association with BD. This report is also comparable with the previous study in which the injunctive norm was negative significant but a weak predictor of BD [17]. Being knowledgeable about BD was also found to be a significant predictor of BD and it implies that an increase in participant’s knowledge on BD would decrease their engagement in BD. This finding supports the assumption of IBM which considers knowledge as an important factor for behavior to occur.

Using cross-sectional study design which fails to report causal inferences is the main limitation of the current study. Although prospective study design is recommended when IBM is used as a conceptual framework to measure the intention and the behavioral performance the current study did not employ that this research design due to a lack of resources and time.

## Conclusion

The finding of the current study demonstrated high prevalence of engagement in BD among University students compared to WHO report. Only experiential attitude, instrumental attitude, and self-efficacy are IBM constructs which had association with intention to BD. BD was associated with experiential attitude, injunctive norm, environmental constraints, and BD knowledge. Hence, this study can serve as a baseline for the designing of intervention that focuses on improving University student’s knowledge about BD and its consequence. It is also important to implement behavior change communication strategies that focus on identified behavioral factors. Furthermore, prospective study should be done using measures of behavioral intention and the behavioral performance at two separate points in time.

## Supporting information

S1 File Dataset of the study. (XLSX)

S2 File Operational definition of variables. (DOCX)
