# Supplementary material for: Predicting binge drinking among university students: Application of integrated behavioral model
Source: PLoS One. 2021 Jul 9;16(7):e0254185. doi: 10.1371/journal.pone.0254185 (PMC8270465; doi:10.1371/journal.pone.0254185)
Supplement: S2 File — (DOCX) [file pone.0254185.s002.docx]

## Operational definitions of study variables

**Binge drink**: The students were asked if they had any drink in the last two weeks and if they had said yes, male were asked if they had 5 drinks for 2 hours in row and female were asked if they had 4 drinks for 2 hours in row. If male student had five and female had four drinks in about 2 hours during the last two weeks of study period, the student is engaged in binge drinking.

**Intention:** individual belief and report of the probability that he/she will binge drink and obtained by sum of statements related to intention which scored using 5 point Likert-scale after reversely scoring negatively worded statement.

**Experiential attitude**: it is individual belief in binge drink is associated with his/her feeling and obtained by sum of statements related to this attitude which scored using 5 point Likert-scale after reversely scoring negatively worded statement.

**Instrumental attitude**: it is individual belief in binge drink is associated with certain out come and obtained by sum of statements related to this attitude which scored using 5 point Likert-scale after reversely scoring negatively worded statement.

**Injective norm**: it is individual belief whether his important persons approves or disprove his/her binge drink and obtained by sum of statements related to this norm which scored using 5 point Likert-scale after reversely scoring negatively worded statement.

**Descriptive norm**: it is individual belief whether his/her important individual binge drink and obtained by sum of statements related to this norm which scored using 5 point Likert-scale after reversely scoring negatively worded statement.

**Self-efficacy-** it is individual confidence toward binge drinking and obtained by sum of statements related to confidence which scored using 5 point Likert-scale after reversely scoring negatively worded statement.

**Perceived control-** it is individual belief on what control him/her from binge drinking and obtained by sum of statements related to control belief which scored using 5 point Likert-scale after reversely scoring negatively worded statement.

**Knowledge**: measured by summing the statement related to knowledge about binge drink after reversely scoring the incorrect statements.
